# Assessing the Professional Development Needs of Public Health Educators in Light of Changing Competencies

**Published:** 2008-09-15

**Authors:** Anne Roesler Demers, Edward Mamary

**Affiliations:** San Jose State University; San Jose State University, San Jose, California

## Abstract

**Introduction:**

Because of the need for a well-trained public health workforce, professional competencies have been recently revised by the Institute of Medicine and the National Health Educator Competencies Update Project. This study compared the self-identified training needs of public health educators with the updated competencies and assessed employer support for continuing education.

**Methods:**

A convenience sample of public health educators was recruited from an e-mail list of San Jose State University master of public health alumni. Respondents completed a Web-based survey that elicited information on emerging trends in public health education, training needs, and employer support for continuing education.

**Results:**

Concerns about funding cuts and privatization of resources emerged as a theme. Key trends reported were an increase in information technology, the need for policy advocacy skills, and the importance of a lifespan approach to health issues. Primary areas for training were organization development, evaluation, and management. Although most employers were reported to support continuing education, less than two-thirds of respondents were reimbursed for expenses.

**Conclusion:**

These findings have implications for both research and practice. Innovative technologies should be developed to address health education professionals' training needs, and emerging themes should be incorporated into curricula for students.

## Introduction

A number of challenges to public health — including war (1), environmental inequalities (2), bioterrorism (3), global warming, globalization, increased travel, and drug-resistant strains of emerging and reemerging diseases — which contribute to accelerated disease transmission (4), greater health disparities between the rich and the poor (5), and food security issues (6) will require complex approaches and will raise crucial ethical, legal, and social questions (7). Therefore, the public health workforce must be educated about these issues and trained to respond effectively. This need for education and training has already been acknowledged by a variety of health-related organizations, including the Institute of Medicine (IOM); American Association of Health Education; American Public Health Association, Public Health Education and Health Promotion Section; Society for Public Health Education; Council on Education for Public Health; and National Commission for Health Education Credentialing.

Because competency requirements for public health practice are not static, the IOM report *Who Will Keep the Public Healthy?* (7) identified 8 emerging areas for competency development: informatics, communications, community-based participatory research, global health, ethics, genomics, cultural competency, and policy and law. Acknowledging that education in these areas is key to enhancing the professional development of health educators, the National Health Educator Competencies Update Project (NHECUP) (8) conducted a survey to assess health educators' practices and to validate advanced-level competencies. The survey identified key areas of responsibility for health education practitioners: assess individual and community needs; plan and implement health education strategies, interventions, and programs; conduct evaluation and research related to health education; administer health education strategies, interventions, and programs; be a resource for health education; and communicate and advocate for health and health education.

Workforce competency is 1 of the core underpinnings of public health infrastructure (9); it enables the public health system to prepare for and respond to both acute and chronic threats to public health. The national focus on emergency preparedness highlights the need for ongoing competency development within the public health workforce, frontline workers and health educators alike. The leaders of the public health community realize that an adequately prepared workforce requires long-term development (10), and public health department leaders have listed retaining and retraining personnel as the highest priorities for attention (11). However, educational competencies used in formal academic settings may not perfectly relate to workforce competencies, which describe the skills, knowledge, and abilities of practitioners in their functional roles (9).

The National Public Health Performance Standards (12) propose that local health departments adopt "continuous quality improvement and lifelong learning programs for all members of the public health workforce, including opportunities for formal and informal public health leadership development." However, little systematic information exists regarding the extent to which local health departments actually use various sources of education and training, which courses and topics are most frequently sought, or the effectiveness of alternative sources of training (7).

The 2003 IOM report (7) acted as a catalyst for the San Jose State University (SJSU) master of public health (MPH) core faculty to review the curriculum for consistency with the content areas recommended by the report for all public health professionals. Later, as part of the criteria for our Council on Education for Public Health accreditation self-study, our MPH program needed to subscribe to specific community health education competencies that were accepted across the profession. We chose those competencies that emerged from NHECUP (8) and were endorsed by the National Coalition of Health Education Organizations as the basis of the core competencies for the health education specialization in the MPH curriculum. The faculty selected the advanced competencies from the NHECUP report to assist in preparing students for the coming master's advanced certification exam. To assess the fit between the IOM competencies (7), the newly adopted MPH program competencies (8), and current issues identified by health education professionals (primarily in the San Francisco Bay area), we conducted a pilot study among SJSU MPH alumni to assess their training needs and employer support for continuing education and identify emerging trends and issues in public health.

## Methods

### Procedure

A 47-question survey, based on the advanced-level competency areas, was developed by the SJSU MPH Program Professional Development Working Group, a 7-member team composed of faculty, community health educators, alumni, and students. The team was established to ensure that the MPH program curriculum addresses the needs of health education professionals in the field. Institutional review board approval was not sought because this survey was incorporated into the routine data collection for our accreditation self-study. Convenience sampling was used to recruit participants for this online pilot survey, which was announced by e-mail to all SJSU MPH alumni on the e-mail list (N = 187). All alumni are added to the list when they graduate, and although they can unsubscribe at any time, less than 10% do so (D. Perales, written communication, December 28, 2007). The alumni list comprises health educators, many of whom hold key positions in a wide variety of public health settings and who are employed primarily in California.

### Questions

We solicited information about the year participants entered and graduated from the MPH program and place of employment (nonprofit organization, university or research, hospital or health care setting, government, proprietary organization, field unrelated to health, or unemployed). Two questions asked participants to identify workforce trends that would be emerging in the next 2 years and in the next 5 years. Participants were then asked if they were required to take continuing education units (CEUs); if so, participants were asked to identify what type of certificate or license they held and to indicate where they complete CEUs. Participants were also asked to identify their education needs from categories such as grant writing, evaluation, management/supervision/administration, ethical issues, social epidemiology, health literacy, organizational development, working with diverse populations, and social marketing. Participants could also identify education needs that were not included in the presumed categories.

One rating question asked participants how well prepared they felt for their current job on a scale of 1 (poor) to 5 (excellent). The remaining questions assessed participants' ability to take time off from work to attend continuing education events, perception of whether employers would pay for CEUs, and logistic questions about preferred days, times, and delivery methods for CEUs.

The survey was launched by Zoomerang.com (Market Tools, Inc, San Francisco, California) on June 20, 2006, and data were collected for 3 weeks. Descriptive data analysis was conducted in SPSS (SPSS Inc, Chicago, Illinois).

## Results

### Description of participants

Seventy respondents (37% response rate), who graduated between 1978 and 2006, completed the survey; almost half (43%) had graduated within the previous 3 years. Ninety-five percent of respondents were employed in health-related fields. Most were employed in government settings, including local health departments (36%); the remainder were employed by nonprofit agencies (24%), universities or research facilities (17%), hospital or health care settings (14%), or proprietary organizations (consulting, pharmacy, or other for-profit companies) (4%). The remaining participants worked in fields unrelated to health or were unemployed at the time of the survey.

### Emerging trends in public health

Survey participants were asked to predict emerging trends in public health education in the next 2 to 5 years; many respondents noted that several key trends would continue to be issues well beyond this period. The top 3 trends that respondents felt affected public health education were 1) improvements to and increasing availability of information technology, which can be used to deliver health education programs and to plan and evaluate these programs (33%); 2) the need to improve policy advocacy skills among health educators (15%); and 3) the importance of a lifespan approach to health issues, given the aging population with which health educators work (13%). A primary theme, linked to trends and identified by 24% of respondents, was concern about continuing cuts in government funding and privatization of resources. Other themes identified were disaster preparedness (11%), environmental health (9%), systems change (7%), community organization (4%), social marketing (4%), and media advocacy (4%).

### Training needs

The top 3 areas identified were 1) organizational development, particularly gaining a better understanding about systems operations, 2) building collaborative efforts across private- and public-sector organizations, and 3) skills to effect change within organizations; evaluation; and management and supervision, specifically management training at both entry and advanced levels (Table). This response is related to the perception that staff will be downsized and remaining employees must take on additional responsibilities. Participants also identified the following specialized areas within management training: project management, time management, fiscal/budget management, personnel management (eg, conflict resolution, ethical approaches to supervising, managing culturally diverse workforces, performance evaluations, interviewing prospective employees, and working with volunteers), and leadership. Participants recognized a need for training in management theory and expressed a strong desire for training in ethical leadership, coaching, and mentoring other professionals.

### Employer support for continuing education

Thirty-one percent of respondents reported that they are required to take CEUs to maintain professional certification or licensure. Seventy-seven percent of those reported that they are certified health education specialists; the remaining respondents reported taking CEUs to maintain certification in the American College of Sports Medicine or in industrial ergonomics or to maintain licensure as registered nurses, medical doctors, or physician assistants. Although 93% of respondents reported that their employers would allow them to take time off work to attend professional development training, 38% (including 11% who are required to maintain health education specialist certification) stated that their employers either do not pay for continuing education or reimburse only limited amounts. The primary reasons cited were budget constraints, cost of travel to conferences, and reimbursement only of training with direct job application.

## Discussion

The trends and training needs identified by participants fall into 5 of the 8 new priority areas described in the IOM report (7) — informatics, communication, policy, cultural competence, and ethics — and into 3 of the 5 competency areas described by NHECUP (8) — conduct evaluation related to health education; administer health education strategies, interventions, and programs; and communicate and advocate for health and health education. The trends and training needs that were identified overlapped substantially, and 3 interconnected areas emerged.

The first area is the need for training in policy advocacy skills and organization development, specifically changing systems, and the need for collaborative efforts within and between health agencies. This training should address complex contemporary health issues and help ensure that appropriate and adequate programs and services are available and accessible to all populations who need them. These needs are most likely being driven by government cutbacks in funding, downsizing the public health workforce, and privatizing resources. The second area is the need for training in informatics, communication, and evaluation. Respondents noted that training in evaluation was a priority and emphasized the need to use information technology in program planning, delivery, and evaluation.

The third area is the need for training in cultural competence, ethics, and administration. The emphasis on leadership, mentoring, and a hierarchical approach to management training (entry level, midcareer, and advanced level) shows that respondents anticipate taking on additional responsibilities. Respondents identified cultural competence and ethics in the context of management and leadership — they indicated a need for training in ethical approaches to supervising and managing a culturally diverse workforce — not in the context of working with populations. Our respondents were all SJSU alumni whose training included cultural competence and ethics in planning, implementing, and evaluating community-based health education programs, and they may not have viewed these skills as transferable to their workplace environments. This finding shows that training in ethics should be incorporated into health care management courses; this training would ideally move beyond the model of business ethics and emphasize a systems approach to ethics in the workplace.

An additional trend identified by participants, but not included in either IOM or NHECUP reports, was the need to frame public health issues from a lifespan perspective. As baby boomers age and more multigenerational families seek health care, public health professionals must treat health and illness as cumulative products of various interacting diseases and conditions, in the contexts of people's lives. Addressing complex health issues will require moving beyond the biomedical model and embracing a paradigm that integrates multiple models, not only those involving the lifespan but also those that include geographic, environmental, and community contexts.

Given the economic circumstances described by respondents, that 38% of employers do not pay for continuing education is not surprising. Although leaders of the public health community may realize that an adequately prepared workforce requires long-term development (10), and public health department leaders have listed retaining and retraining personnel among their highest priorities (11), many of them cannot afford to do what is in their own best interests and those of the communities they serve.

### Limitations

The use of convenience sampling resulted in a self-selected group of participants, and our findings may not be generalizable to all public health educators. This study would have been strengthened by the inclusion of 3 additional groups: 1) public health educators from MPH programs other than that of SJSU, 2) public health educators with a bachelor's degree, and 3) public health professionals without MPH or bachelor's degrees but with work experience in public health education (eg, health promoters or lay health educators). Future assessment should include these groups to more accurately identify the training needs of health educators at all levels of employment.

### Conclusions

By identifying priority training areas, the results of this survey provide a basis for conducting a more systematic assessment of health educator needs. The training needs identified by the participants in this study are in line with updated health education competencies (8) and a number of the emerging areas documented by the IOM (7). However, in light of diminishing resources, universities must seek ways to collaborate with organizations to offer ongoing training for public health educators and seek ways to deliver cost-effective training via the Internet (13). In addition to identifying training needs for the current workforce, the themes identified through this research — workplace ethics, a lifespan approach, organization development, management and supervision, coalition building, information technology, and policy advocacy — once validated by a more systematic assessment process, should be considered when planning curricula so that students are adequately prepared to assume leadership positions in health education. Finally, universities must continue to collaborate with health education professionals in the field, not only to provide training programs but also because they are uniquely positioned to identify emerging issues and changing trends in public health. Their insights are crucial to informing the education required to effectively address the complex issues faced by the public health workforce.

## Figures and Tables

**Table:** Table. Training Needs for Public Health Education Professionals Identified by Alumni of the San Jose State University Master of Public Health Program, 2006

**Training Need**	**%**
Organizational development	43
Evaluation	41
Management	41
Grant writing	21
Nonprofit administration	21
Social epidemiology	20
Social marketing	16
Disaster preparedness	13
Health literacy	12
Materials development	8
Diverse populations	8
Ethical issues	4
